# When the Clock Shifts: A Comprehensive Review of Daylight-Saving Time (DST), Circadian Disruption, and Neuropsychological Risk in Chronic Mental Illness

**DOI:** 10.3390/brainsci16050522

**Published:** 2026-05-14

**Authors:** Liahm Blank, Joshua Khorsandi, Elizabeth England-Kennedy, Srikanta Banerjee, Karen Kopera-Frye, Roberto Sagaribay, Jagdish Khubchandani, Kavita Batra

**Affiliations:** 1 Department of Medical Education, Kirk Kerkorian School of Medicine at UNLV, University of Nevada Las Vegas, Las Vegas, NV 89102, USA; blankl1@unlv.nevada.edu (L.B.);; 2 College of Health, Education, and Social Transformation, New Mexico State University, MSC 3AC, P.O. Box 30001, Las Cruces, NM 88003, USAkfrye@nmsu.edu (K.K.-F.);; 3 College of Health Sciences, Walden University, Minneapolis, MN 55401, USA; 4 Office of Research, Kirk Kerkorian School of Medicine at UNLV, University of Nevada Las Vegas, Las Vegas, NV 89102, USA

**Keywords:** daylight saving time (DST), sleep disruption, cognition, dysregulation, mental illness, circadian rhythm

## Abstract

**Highlights:**

**What are the main findings?**

- Daylight Saving Time (DST) transitions act as a population-wide circadian stressor, leading to sleep disruption, cognitive impairment, emotional dysregulation, and short-term increases in psychiatric symptoms, including depression, anxiety, and suicidality.
- Individuals with chronic mental illness appear particularly vulnerable to DST-related circadian misalignment due to underlying biological and neuropsychological susceptibilities, including disruptions in melatonin, cortisol, and clock-gene regulation.

**What are the implications of the main findings?**

- DST transitions should be recognized as predictable, modifiable risk periods in clinical practice, warranting anticipatory guidance, sleep stabilization strategies, and closer monitoring of high-risk psychiatric populations.
- At the population level, findings support growing calls to reconsider or eliminate seasonal clock changes, as DST may represent a preventable environment risk factor for adverse mental health outcomes.

**Abstract:**

Daylight Saving Time (DST) creates abrupt, externally imposed circadian disruptions that can impair sleep regulation, hormonal balance, cognitive performance, and emotional stability. Although these effects are known in the general population, individuals with chronic mental illness, whose circadian systems are often intrinsically dysregulated, may face increased neuropsychological consequences. This comprehensive review synthesizes evidence from chronobiology, psychiatry, neuroscience, and population health to examine how DST-related circadian misalignment impacts cognitive functioning, mood regulation, suicidality risk, and symptom exacerbation across psychological disorders such as depression, anxiety disorders, bipolar disorder, post-traumatic stress disorder, attention-deficit/hyperactivity disorder, and psychotic disorders. Following the Scale for the Assessment of Narrative Review Articles (SANRA) guidelines, a search of PubMed, PsycINFO, Scopus, and Google Scholar was conducted to identify studies published from 2000–2026 examining DST, circadian rhythm disruption, neuropsychological outcomes, and chronic mental illness. Empirical, theoretical, and mechanistic studies were included to ensure comprehensive synthesis. Across conditions, DST, particularly spring forward transitions, is associated with increased sleep disturbance, impaired executive functioning, reduced attention and working memory, heightened emotional reactivity, increased depressive symptoms, elevated risk of manic episodes, and short-term increases in suicidality. Neurobiological mechanisms include altered melatonin secretion, cortisol dysregulation, Hypothalamus Pituitary Axis (HPA-axis) activation, and clock-gene desynchrony. DST may function as a modifiable negative environmental influence capable of affecting neuropsychological functioning in vulnerable populations. These findings underscore the need for clinical awareness, preventive strategies, and policy reconsiderations, including calls to eliminate seasonal time changes. Standardizing DST-related research outcomes and expanding longitudinal, multi-site studies will be essential for advancing this emerging field.

## 1. Introduction

Daylight Saving Time (DST) is a seasonal practice involving the advancement of clocks by one hour in the spring (“spring forward”) and their return to standard time in the fall (“fall back”). Originally implemented to conserve energy and optimize daylight use, DST is currently observed in over 70 countries worldwide, including large portions of North America and Europe, affecting more than one billion people annually [1]. While the necessity of DST in the modern era is contested, critics of the continuation of the practice cite that the biannual clock changes impose an abrupt, externally mandated alteration to social and biological timing systems: DST changes the timing of daylight, which is a major regulator of the circadian rhythm in humans [2].

Though these one-hour shifts in the light–dark schedule may seem trivial, growing evidence suggests that these transitions may act as a population-wide circadian stressor, with implications for health and safety [3,4]. For example, epidemiologic studies have linked DST, particularly the spring transition where an hour of sleep is lost, to increased risks of cardiovascular events [5], workplace [6] and traffic accidents [7], and all-cause mortality in the days following clock changes [4,8]. Emerging evidence further suggests that DST may be associated with increased healthcare utilization, including emergency department visits and hospital admissions [9], primarily due to negative health outcomes.

Although many disruptions to bodily system functioning have been noted, sleep disruption is one of the most consistently observed effects of DST. Sleep-related terminology is often used interchangeably in the literature, but important distinctions exist. Sleep deprivation refers specifically to insufficient total sleep duration, whereas sleep disturbance encompasses a broader range of disruptions. In contrast, circadian misalignment refers to a mismatch between endogenous biological rhythms and external environment or social cues. In this comprehensive review, DST is conceptualized as an externally imposed circadian perturbation that may contribute to acute sleep loss and broader sleep disturbances, which in turn may influence psychiatric outcomes. Certain subpopulations may be particularly vulnerable to DST-related circadian disruption, including adolescents and students, who frequently exhibit irregular sleep–wake patterns, chronic sleep restriction, and heightened academic or social demands, all of which may exacerbate the impact of abrupt clock changes.

Multiple studies demonstrate reductions in total sleep time, increased sleep disturbance, and disruption of natural circadian rhythms following DST transitions across populations, with incomplete adaptation in many individuals [10,11,12]. Importantly, sleep and circadian disturbances are not merely benign inconveniences; they are well-established contributors to emotional imbalance, cognitive impairment, and higher stress reactivity [13]. Sleep loss and circadian disruption have been implicated in impaired executive functioning, heightened negative affect, and reduced coping capacity, all of which are particularly relevant to wellbeing and mental health [14,15].

Although much of the literature emphasizes the adverse effects of DST transitions, it is important to acknowledge that these effects are often transient. While many individuals adapt to DST-related circadian disruption within several days, evidence suggests that the timeline of adjustment is variable. In the general population, sleep and circadian realignment often occurs within approximately 2–7 days following the transition. However, in individuals experiencing greater circadian misalignment, adjustment may take up to one to two weeks [10]. Importantly, in populations with underlying psychiatric or circadian vulnerabilities, adaptation may be prolonged, incomplete, or characterized by ongoing instability in sleep and symptom patterns [10,11]. Additionally, DST has been associated with potential benefits, including increased evening daylight, which may promote physical activity, social engagement, and outdoor exposure. However, these potential advantages do not necessarily offset the short-term disruptions in sleep and circadian alignment observed immediately following the transition, particularly among vulnerable populations.

Sleep and mental health are closely interconnected in a bidirectional relationship. Disturbances in sleep and circadian rhythms are core symptoms of depressive disorders, bipolar disorder, anxiety disorders, post-traumatic stress disorder (PTSD), attention-deficit/hyperactivity disorder (ADHD), and psychotic disorders [16,17,18,19]. Experimental sleep deprivation studies and longitudinal observational research have shown that even short-term sleep loss can precipitate mood destabilization, increase anxiety symptoms, impair attention and cognitive function, and elevate suicidal ideation in vulnerable individuals [18,20,21]. For instance, in bipolar disorder, circadian disruption is strongly associated with manic relapse [22], while in PTSD, sleep disturbance has been linked to maladaptive stress, coping, and trauma responses leading to poor psychiatric outcomes [23]. At the same time, psychiatric conditions themselves frequently disrupt sleep through mechanisms such as heightened arousal, dysregulated stress response, and altered circadian signaling [24,25]. This reciprocal interaction can create a self-reinforcing cycle in which sleep disruption and psychiatric symptoms exacerbate one another over time. Within this framework, DST-related circadian disruption may act as an initial perturbation that destabilizes both sleep and mental health, particularly in vulnerable populations.

Despite these converging lines of evidence, the literature examining DST, sleep disruption, and mental health remains fragmented and limited in scope. Research on DST has largely focused on physical health outcomes, accidents, or population-level mortality, often without consideration of psychiatric vulnerability or neuropsychological functioning [5,6,8]. Likewise, the extensive literature on sleep and circadian dysregulation in mental illness rarely explores these mechanisms within the context of DST or seasonal time changes [16,18]. As a result, key domains of research, such as DST epidemiology, sleep and circadian biology, and psychiatric vulnerability, have developed largely in parallel rather than in an integrated manner.

The literature on DST, circadian disruption, and mental illness includes a variety of methodological approaches, including several cross-sectional surveys capturing the association of sleep disturbances and circadian dysregulation with symptom severity, level of functioning, self-harming behaviors, and benefit from treatment across different mental illnesses [26,27,28]. These complementary survey-based studies and clinical samples highlight associations between sleep disturbance, circadian rhythm instability, cognitive and emotional dysregulation, and symptom severity across mood, anxiety, and psychotic disorders. However, findings were also variable due to heterogeneity in study design, reliance on self-reported measures, variability in diagnostic definitions and outcome assessments, and constraints on causal inference [26,27,28].

In addition to survey studies, many observational studies on circadian disruption and mental illness exist. These studies help support temporal and mechanistic associations between sleep dysregulation and mental illnesses such as bipolar disorder, major depressive disorder, and schizophrenia, underscoring how DST may contribute to symptom exacerbation [28,29,30]. Many of the included studies employed diverse validated instruments to assess sleep and psychiatric symptomatology, including clinician-rated scales such as the Hamilton Rating Scale for Depression and the Montgomery–Åsberg Depression Rating Scale, as well as self-report measures like the Pittsburgh Sleep Quality Index and the Horne–Östberg Morningness–Eveningness Questionnaire [28,29,31]. Other studies employed mixed methods to assess circadian rhythms and chronotypes (i.e., individual differences in preferred timing of sleep and activity across the 24-h cycle) in relation to mental illness, utilizing cross-sectional data, surveys, and objective biological measurements (e.g., plasma or saliva levels) [29,32]. Some of these studies utilized actigraphy, a noninvasive, objective method for measuring sleep–wake patterns, activity levels, and circadian rhythms using a small wearable device [21,30,33]. While these studies are less generalizable due to smaller sample sizes, they provide additional objective contributions to the presumed relationship between sleep disruption and mental illness exacerbation that cannot be fully captured by cross-sectional survey studies.

Furthermore, systematic and scoping reviews have provided an important synthesis of the overall themes encompassing DST, circadian disruption, and mental illness. Reviews demonstrate that circadian rhythm dysregulation and chronotype (preferred sleep timing) differences are key features of several psychiatric disorders, such as bipolar disorder, ADHD, schizophrenia, and major depressive disorder [21,34,35,36]. The reviews further underscore the role of an individual’s physiology in mental illness by summarizing the genetic and neural pathways that are thought to contribute to exacerbated symptomology by interacting with the body’s circadian rhythm [24,37,38,39]. Therefore, this comprehensive review synthesizes the existing literature and research to date, examining the interplay between DST, circadian rhythm dysregulation, and psychiatric vulnerability while highlighting gaps that remain in understanding disorder-specific mechanisms and population-level mental health impacts.

Across studies, the literature demonstrates that different methodological approaches contribute complementary strengths to the evidence base. Epidemiologic and survey studies offer broad population-level insights, observational studies support associated relationships, and reviews allow the integration of multiple lines of evidence. Together, these multiple methods highlight the importance of synthesizing findings across fields of study to understand how DST-related circadian disruption may influence psychiatric disturbance among vulnerable individuals. The diverse body of literature thus contributes to the increasing awareness of the impact of DST on individuals with mental illness.

Given the extensive exposure to DST among individuals and the well-established links between sleep disruption and psychiatric outcomes, a comprehensive synthesis of this literature was warranted. The present comprehensive review has two primary aims. The first aim is to synthesize existing evidence on the relationships among DST, sleep and circadian disruption, and mental health outcomes, with particular attention to individuals with chronic mental illness. The second aim is to investigate the potential neurobiological mechanisms underlying heightened vulnerability to DST-related circadian disruption in individuals with mental illness. By integrating epidemiologic, clinical, and biological pathway-based evidence, this review seeks to explore how DST may contribute to neuropsychological disturbance and psychiatric destabilization, and to identify directions for future research, clinical practice, and public health policy.

This comprehensive review was guided by two primary research questions to examine the population-level impacts of Daylight-Saving Time and the underlying mechanisms linking circadian disruption to mental health vulnerability.

1. How does Daylight Saving Time-related circadian disruption impact cognitive functioning, mood regulation, suicidality risk, and symptom exacerbation among individuals suffering from chronic mental illnesses, including depression, bipolar disorder, anxiety disorders, PTSD, ADHD, and psychotic disorders?
2. What potential neurobiological mechanisms underlie the impacts of DST-related circadian disruption among individuals with chronic mental illness?

## 2. Methods

### 2.1. Literature Search

This comprehensive review was conducted from 16 December 2025 through 27 February 2026 in accordance with the SANRA (Scale for the Assessment of Narrative Review Articles) guidelines [40]. A comprehensive approach was used to synthesize evidence across DST, sleep disruption, and mental health research, integrating population-level outcomes with insights from biological pathways relevant to psychiatric vulnerability. Because DST-specific literature is limited, mechanistic and disorder-specific circadian studies were also included to support biological plausibility and integrative interpretation. To capture the breadth of literature, PubMed, Embase, Web of Science, and Scopus were searched and restricted to English language-only studies published from 2000 to 2026. Table 1 displays the search terms used for each database and the number of articles yielded from each database. The reference lists of key papers were cross-analyzed to identify additional papers. In total, the search yielded 1556 studies across the four databases before screening.

### 2.2. Inclusion and Exclusion Criteria

Studies that were included are those that: (a) were published in peer-reviewed journals; (b) included analyses focusing specifically on Daylight Saving Time (DST), seasonal time changes, or seasonality in relation to mental health illness or psychiatric outcomes; (c) included analyses focusing specifically on circadian rhythm disruption, circadian misalignment, or sleep disruption relevant to psychiatric functioning; (d) focused on individuals with chronic mental illness, including those suffering from depression, bipolar disorder, anxiety disorders, post-traumatic stress disorder (PTSD), attention-deficit/hyperactivity disorder (ADHD), psychotic disorders, or populations at elevated psychiatric risk; and (e) were empirical, mechanistic, or theoretical analyses that informed integrative synthesis of DST-related biopsychosocial risk. Sources excluded were those that: (a) did not directly analyze mental health, psychiatric, or neuropsychological outcomes in relation to DST, seasonality, or circadian rhythm disruption; (b) only examined non-psychiatric medical outcomes of circadian disruption (e.g., cardiovascular or metabolic conditions) without mental health relevance; or (c) focused solely on evaluating the effects of a single psychiatric treatment in relation to sleep. Screening and selection were done by one author. Two-hundred and sixty-three articles were included at the abstract screening stage; 66 of those articles met the inclusion criteria at the full text stage and were included in this review. Additional relevant articles identified during manuscript preparation were also included in this review (e.g., authoritative sources such as clinical guidelines were consulted to inform clinical and policy implication sections).

### 2.3. Analytical Approach

**Figure 1:**
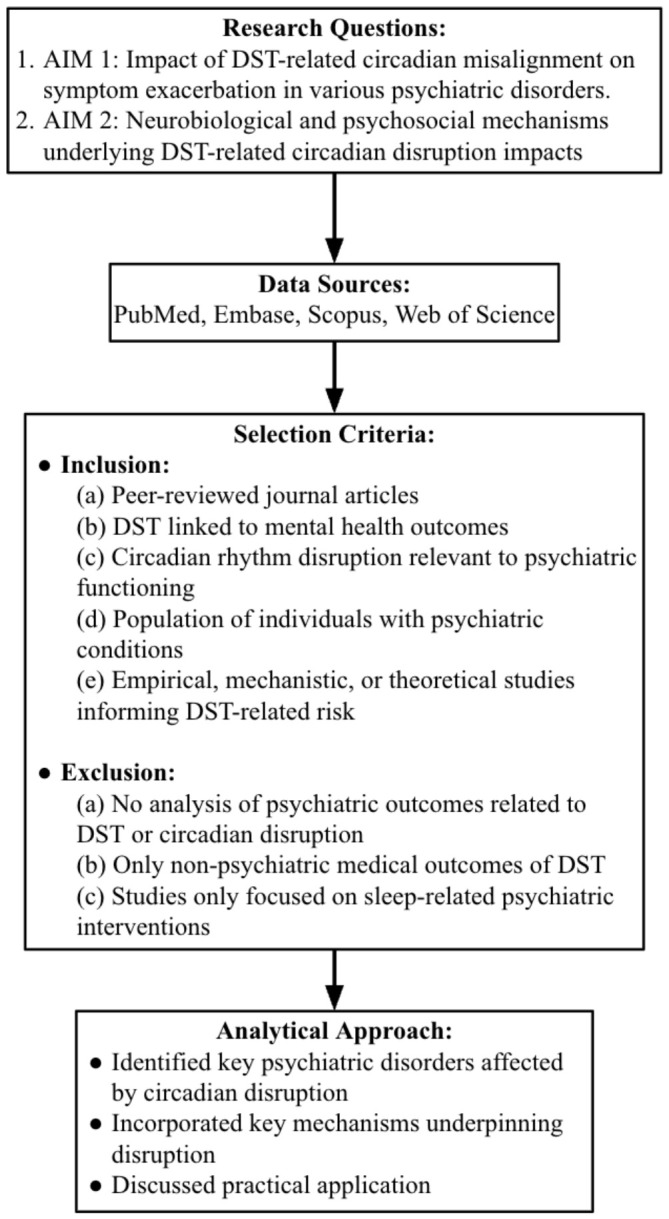
Conceptual framework guiding the comprehensive review. The figure depicts the overarching structure of the review, outlining the research questions, the literature sources, eligibility criteria, and integrative analytic strategy. It illustrates how evidence from multiple data sources was systematically identified and synthesized to address the review aims.

The findings of this comprehensive review are synthesized categorically based on the two aims (Figure 1). For the first aim, the subsections are based on the major psychiatric disorders that encompass circadian dysfunction as a key feature, as identified by the literature: (1) depressive disorders (2) anxiety disorders, (3) bipolar disorder, (4) post-traumatic stress disorder, (5) attention-deficit/hyperactivity disorder, and (6) psychotic disorders, including schizophrenia. For the second aim, the subsections are derived from studies focused on elucidating the underlying neurobiological mechanisms involved in the relationship between sleep dysfunction and psychiatric morbidity.

In resolving these aims, the results are derived from a diversity of study designs, including cross-sectional surveys, observational studies, mixed-methods research, and systematic reviews, primarily featuring populations diagnosed with mental illness. A future reaching approach then relates findings to practical significance, including implications for clinical practice and policy. Accordingly, this review integrates evidence across levels of analysis, linking individual-level findings with neurobiological mechanisms and broader social influences.

This synthesis encompasses several methodological strengths. First, the cross-sectional surveys included in this review offer limited evidence of associations between DST-related sleep disturbance and psychiatric vulnerability, while observational studies suggest possible associations between DST and exacerbation of psychiatric symptomology. Second, the inclusion of systematic reviews allows for broader, though methodologically diverse, integration of the available literature. As a function of incorporating evidence across all these methods, this review has the potential to emphasize not only what is known about DST-related psychiatric vulnerability, but also the research practices shaping the findings. The SANRA guidelines were used to guide the development of this review, ensuring clear justification of the topic’s importance, well-defined aims, a thorough literature search, and rigorous scientific reasoning grounded in evidence.

To improve interpretability, included studies were grouped into three categories based on their relevance to the research question: (1) DST-specific epidemiological studies, which directly examine outcomes associated with DST transitions; (2) Observational studies of sleep and psychiatric outcomes, which provide indirect evidence by examining the relationship between sleep or circadian disruption and mental health; and (3) Mechanistic and experimental studies, which explore underlying biological processes such as melatonin regulation, HPA-axis activity, and clock gene expression. This classification was used to distinguish between direct and indirect evidence when synthesizing findings. Of the 66 studies included in this review, approximately 12 were DST-specific epidemiological studies (direct evidence), 36 were observational studies examining sleep and psychiatric outcomes (indirect evidence), and 18 were mechanistic or experimental studies exploring underlying biological pathways.

## 3. Results

Direct DST-specific evidence for this condition is limited; therefore, much of the following discussion draws on indirect evidence from sleep and circadian research. DST-related sleep disruption may affect multiple dimensions of sleep, rather than a single uniform outcome. These include reduced sleep duration, particularly following the spring transition, as well as increased sleep fragmentation and decreased sleep continuity, which may impair overall sleep quality. Additionally, DST may influence sleep architecture, particularly the balance between non-rapid eye movement (NREM) and rapid eye movement (REM) sleep. REM sleep predominates in the latter portion of the sleep cycle and plays a critical role in emotional regulation, memory consolidation, and processing of affective experiences [41]. Because the spring transition to DST effectively advances wake time, it may lead to truncation of REM-rich sleep periods, even when total sleep loss appears modest. In contrast, NREM sleep, particularly slow-wave sleep, is more likely to be preserved due to its prioritization earlier in the night. This selective reduction in REM sleep may contribute to short-term impairments in emotional regulation and cognitive functioning, providing a plausible mechanism linking DST-related sleep changes to psychiatric symptom exacerbation. Beyond clinical outcomes, DST-related sleep disruption may also have broader functional implications. Even modest reductions in sleep duration may lead to increased fatigue, reduced alertness, and impaired cognitive performance, which may translate into decreased workplace productivity and a higher likelihood of errors [13,14,15]. Prior research has linked sleep loss to attentional lapses and slower reaction times, outcomes that may be particularly relevant in safety-sensitive occupations. Although these effects are less transient, they highlight the potential for DST transition to influence not only mental health but also day-to-day functioning and performance.

### 3.1. DST and Psychiatric Risk

#### 3.1.1. DST, Circadian Dysfunction, and Depressive Disorders

Depression has been consistently linked to disturbances in sleep and circadian regulation [25]. Individuals with depression commonly exhibit altered sleep–wake timing, impaired sleep continuity, prolonged sleep latency, increased intermittent awakenings, early morning awakenings, and dysregulated melatonin and cortisol rhythms [25,26,42]. While sleep disturbance is a known symptom of depression, a substantial body of research further demonstrates that changes in sleep can reciprocally contribute to the development of depression, exacerbate depressive symptoms, and even reduce the efficacy of pharmacological treatment for depression [25,43]. One study observed as internal circadian disruption increased, depressive symptom severity worsened, independent of sleep duration or timing [29]. Other studies demonstrate that even modest sleep loss or circadian disruption may increase symptoms related to depressive disorders, such as negative affect, emotional dysregulation, and risk of suicidal behavior [43,44]. Within this context, DST represents a unique, population-wide circadian perturbation that may significantly impact individuals with depression. Evidence from an epidemiologic study indicates that DST transitions, particularly the autumn shift back to standard time, are associated with an 11% increase in the incidence rate of unipolar depressive episodes [1]. Likewise, one epidemiologic study reports that the sleep disruptions during the Springtime transition may correlate to the suicide rate to rise by 6.25 percent in the United States, most of which practices DST [45]. This is consistent with finding from Berk et al. [46], which reported that even small shifts in diurnal rhythms, such as those associated with DST transitions, were linked to increases in suicide risk. Collectively, this evidence indicates that DST-related circadian disruption may exacerbate depressive symptomatology by intensifying underlying circadian misalignment in this susceptible population.

#### 3.1.2. DST, Circadian Dysfunction, and Anxiety Disorders

Anxiety disorders are closely intertwined with sleep and circadian dysregulation, with difficulties in initiating and maintaining sleep as notable features [47]. Insomnia is significantly associated with several anxiety disorders, including generalized anxiety disorder, agoraphobia, social anxiety disorder, panic disorder, obsessive–compulsive disorder, and PTSD (discussed in its own section below) [27]. Oftentimes, insomnia develops alongside or after anxiety symptoms, but insomnia has been shown to precede the development of anxiety disorders [48]. Because DST has been associated with decreased sleep efficiency and duration in healthy populations [49], it is plausible that these time transitions may converge with circadian dysfunction in anxiety disorders. Furthermore, meta-analytic findings in youth indicate that evening circadian preference is associated with higher anxiety levels [50], suggesting that individuals already displaced later in the day may be particularly sensitive to the phase change imposed by DST. Although DST-specific research on anxiety outcomes remains extremely limited, the convergence of DST-related sleep disruption with established sleep–anxiety mechanisms suggest DST transitions may function as a time-linked stressor that exacerbates anxiety symptoms in vulnerable individuals. Further research is needed to strengthen the understanding of the relationship between anxiety disorders and DST transitions.

#### 3.1.3. DST, Circadian Dysfunction, and Bipolar Disorder

Bipolar disorder is characterized by pronounced dysregulation of sleep and circadian rhythms, with sleep–wake disturbances occurring across mood states and sleep change often serving as a clinically meaningful marker of mood instability [22,33,51]. In particular, insomnia and irregular sleep timing are common: During depressive episodes, individuals with bipolar disorder may experience hypersomnia or severe insomnia, while during manic episodes, individuals frequently experience reduced need for sleep [22,33,34]. Notably, sleep loss is widely recognized as a trigger for manic symptom escalation in susceptible individuals [22,33,51]. Additionally, sleep disturbance among patients with bipolar disorder is significantly associated with the number of previous suicide attempts [28]. It is even postulated that individuals with bipolar disorder have a genetic predisposition to sleep problems, contributing to circadian dysregulation prevalent in bipolar disorder [22]. Thus, circadian stability may be a core target for relapse prevention and favorable prognosis of bipolar disorder [34,51].

Conceptually, predisposition to circadian disruption creates a plausible pathway through which DST transitions could destabilize bipolar illness: for instance, sleep loss is a well-described trigger of manic episodes, and the autumn DST transition may directly precipitate sleep loss [10,22]. However, the small number of studies that have directly tested DST effects on bipolar-relevant outcomes have yielded largely null or mixed findings: An epidemiologic study reported no significant increase in emergency department visits due to mental or behavioral health difficulties, including manic episodes, attributable to DST changes despite inconsistent seasonal variation in presentations [52]. Similarly, a nationwide Finnish registry analysis found that transitions into or out of DST did not significantly increase the incidence of hospital-treated manic episodes in the two weeks after either clock change [53]. This apparent discrepancy may reflect limitations in how psychiatric outcomes are measured in large-scale observational studies. Emergency department and hospitalization data primarily capture severe or acute episodes, and may not be sensitive to subclinical symptom changes, early mood instability, or short-lived increases in manic or hypomanic symptoms. As such, DST-related sleep disruption may still contribute to symptom exacerbation without necessarily resulting in increases in emergency-level care utilization. Taken together, the literature suggests that while DST-related circadian disruption is mechanistically relevant to bipolar vulnerability, its detectable effects on severe manic episodes requiring emergency medical care may be modest or under-researched. Future longitudinal studies on DST and bipolar disorder may elucidate a stronger correlation.

#### 3.1.4. DST, Circadian Dysfunction, and PTSD

Post-traumatic stress disorder (PTSD) is tightly coupled with sleep and circadian dysregulation, with sleep disturbances reported in up to 90% of patients [35]. DST may therefore exacerbate such disturbances. One actigraphical (movement-based sleep monitoring) observational study reported that individuals with PTSD were more likely to experience greater sleep fragmentation, including greater nocturnal activity and increased awakenings [21]. Importantly, disturbed sleep in PTSD is not merely a symptom or result of PTSD: Accumulating evidence indicates that sleep problems may predispose to, precipitate, and perpetuate PTSD [35,54]. For example, circadian disruption before or soon after trauma has been shown to increase the risk for later PTSD development [35,54]. Furthermore, sleep disturbances have been shown to contribute to a worse clinical course for patients with PTSD: in a study of veterans with PTSD, worse baseline sleep quality was associated with less improvement in PTSD symptoms [35,55]. Conversely, targeted sleep interventions have not only been shown to alleviate sleep symptoms, but have also been shown to mitigate daytime PTSD symptoms [35,56]. However, a 2023 meta-analysis reported that less total sleep time was only moderately associated with more severe PTSD symptoms, and bedtime/wake time were not significantly associated with PTSD [57]. The confluence of DST-related sleep disruption and the central role of circadian instability in PTSD suggests DST could plausibly worsen trauma-related insomnia, hyperarousal, and daytime symptom burden during vulnerable post-transition windows, especially for individuals with pre-existing sleep disturbance [35]. However, direct evidence linking DST transitions to acute PTSD symptom exacerbation is extremely limited. Further research is needed to strengthen the understanding of the relationship between PTSD and DST transitions.

#### 3.1.5. DST, Circadian Dysfunction, and ADHD

Evidence directly examining the effects of daylight-saving time (DST) on ADHD is limited; however, related circadian and sleep research suggests that individuals with ADHD may be particularly vulnerable to DST-related disruptions. Individuals with ADHD often experience intrinsic sleep and circadian rhythm disturbances, with an estimated 73–78% showing a delayed sleep/wake cycle, which may render them especially vulnerable to the abrupt one-hour clock shifts in DST [58]. In fact, even modest sleep deficits have been shown to significantly worsen ADHD-associated impairments: One experiment found that after a week of losing just one hour of sleep per night, children with ADHD showed markedly poorer attention (Continuous Performance Test scores) that deteriorated from subclinical to clinically impaired ranges [59]. Similarly, circadian factors play a role in symptom severity: One study reported that later sleep timing and the resultant daytime sleepiness predicted greater ADHD symptom severity independent of total sleep duration [60]. Notably, seasonal shifts in light/dark cycles have been linked to ADHD symptom fluctuations; significantly fewer inattention symptoms have been seen in children with ADHD during summer (longer daylight, DST period) compared to winter, suggesting that robust light-aligned circadian schedules benefit ADHD regulation [61]. Direct DST-specific outcome studies in ADHD are sparse; suggested effects are primarily derived from broader sleep/circadian literature. These findings, however, suggest that DST’s imposed circadian disruptions (especially via sleep loss and phase shifts) may acutely amplify inattention, impulsivity, and mood dysregulation in ADHD individuals by aggravating their already fragile sleep–wake balance.

#### 3.1.6. DST, Circadian Dysfunction, and Psychotic Disorders

Schizophrenia and related psychotic disorders are strongly associated with sleep and circadian rhythm dysregulation, such as delayed sleep onset, impaired sleep continuity, and increased time awake [30,62]. Objective work using prolonged actigraphy indicates that circadian disruption in schizophrenia may be profound even during clinical stability: In one cohort, all participants with schizophrenia demonstrated significant sleep/circadian disruption and roughly half exhibited severe circadian misalignment (e.g., phase advances/delays, highly irregular and fragmented sleep, and non-24-h sleep–wake and melatonin rhythms), despite stable mental state and antipsychotic treatment [30]. A systematic review indicated that sleep disruption among patients with schizophrenia may predict the onset and persistence of psychotic experiences such as paranoia and hallucinations [36]. Some studies note seasonal variations in schizophrenia-related hospitalizations, indicating that decreased exposure to sunlight may be correlated with increased risk of hospital admissions for schizophrenia [63,64]. This relationship highlights the sensitivity of schizophrenia to light-driven circadian changes, suggesting that DST-related shifts in light exposure and timing may similarly contribute to symptom destabilization.

Given the baseline circadian vulnerability in psychotic disorders such as schizophrenia, DST-related changes in sleep timing and light exposure may plausibly exacerbate sleep fragmentation and functional impairment, potentially increasing susceptibility to symptom exacerbation. However, the limited DST-specific outcome literature remains mixed, with early work finding no detectable change in psychiatric admissions for psychotic disorders around DST transitions [65]. Furthermore, an emergency-department study reporting no significant increase in mental/behavioral health visits, including mental illness encompassing psychoses, during the two weeks following DST changes [52]; however, such data may not capture subclinical or short-term symptom fluctuations. Across psychiatric conditions, DST-specific findings remain heterogeneous, and the absence of consistent effects in some studies suggests that DST-related impacts may be modest, context-dependent, or not fully captured by population-level outcome measures.

### 3.2. Mechanisms Underlying DST-Related Psychiatric Exacerbation

#### Specific Brain-DST Pathway Connections

- Light, Suprachiasmatic Nucleus, and Melatonin Secretion

DST does not change the photoperiod (the number of daylight hours), but it does shift the timing of light exposure relative to the social clock—an important issue considering light is a powerful regulator of the human circadian system. Light information from the retina entrains the suprachiasmatic nucleus (SCN), the brain’s central clock, which coordinates downstream rhythms, including nocturnal melatonin secretion from the pineal gland. [66]. This physiological pathway is implicated in psychiatric disorders such as bipolar disorder, where melatonin secretion abnormalities (such as lower baseline melatonin levels) have been observed in experimental studies [67,68]. Other experimental studies have shown that individuals with bipolar disorder in the top tercile of light exposure were significantly less likely to be depressed compared to the bottom tercile [37]. Thus, by decreasing exposure to morning light, DST may increase the risk for circadian misalignment by altering melatonin rhythms, thereby exacerbating symptoms during post-transition adjustment windows in populations with pre-existing vulnerabilities in sleep physiology due to mental illness.

- Hypothalamic–Pituitary–Adrenal (HPA) Axis

Circadian disruption impacted by DST is also biologically significant because the SCN helps regulate the hypothalamic–pituitary–adrenal (HPA) axis, shaping daily rhythms in glucocorticoid (cortisol) secretion that influence arousal, cognition, and stress responsivity [69]. One study observed that sleep deprivation, such as that impacted by DST, may increase cortisol levels and inflammatory cytokine release [32]. Notably, hypercortisolemia has been attributed to dysregulation in negative feedback mechanisms within the HPA axis and is thought to play a central role in the pathogenesis of both depressive symptoms and cognitive deficits pertaining to mood disorders [70]. Evidence indicates that early life trauma induces long-term HPA-axis hyperactivity by sensitizing CRH signaling pathways, leading to persistently elevated cortisol secretion and increased vulnerability to mood and anxiety disorders in adulthood [38]. Thus, by destabilizing cortisol regulation, DST-related sleep disruption may acutely heighten autonomic arousal and emotional reactivity, contributing to symptom exacerbation, relapse vulnerability, and increased psychiatric morbidity in the post-DST period.

- Genetic Susceptibility

Individual differences in vulnerability to DST and circadian disruption likely reflect, in part, variation in the molecular circadian clock. At the cellular level, circadian timing is generated by a transcription-translation feedback loop in which CLOCK and BMAL1 drive expression of *PER* and *CRY* genes, which then inhibit CLOCK/BMAL1 activity to produce 24-h rhythmicity across central and peripheral tissues [24]. Reviews in human genetics and psychiatric genomics suggest that circadian and clock-controlled gene variation contributes to interindividual differences in sleep timing and risk for mental health outcomes, supporting clock genes as plausible candidates for psychiatric vulnerability [71]. For instance, researchers observed a loss of rhythmic expression of *CRY1* and *PER2* in cells of patients with chronic schizophrenia compared to cells from healthy controls [72]. Likewise, in mood disorders, specific variants have been repeatedly investigated: for example, the CLOCK 3111T/C (rs1801260) polymorphism has been associated with bipolar-relevant phenotypes, including increased frequency of manic episodes in some studies [73]. Environmental disruptions may thus interact with clock-gene variation to influence mood regulation, sleep homeostasis, and stress responsivity, influencing pathways directly implicated in psychiatric disorders [39].

DST-related circadian disruption may also be understood within the broader context of other well-established models of circadian misalignment, including shift work and chronotype variation. Shift work, particularly night and rotating schedules, has been consistently associated with increased risk of mood disorders, anxiety, and impaired cognitive functioning, largely due to chronic misalignment between endogenous circadian rhythms and external demands. Similarly, individuals with an evening chronotype often experience misalignment with socially imposed schedules, which has been linked to increased depressive symptoms, anxiety, and poorer mental health outcomes. Although DST represents a more acute and time-limited perturbation, these parallels reinforce the concept that even modest misalignment between biological and social time may contribute to psychiatric vulnerability, particularly in susceptible populations.

### 3.3. Practical Application of DST-Related Circadian Disruption

#### 3.3.1. Clinical Applications

Clinically, DST transitions may be treated as a predictable circadian stressor for patients with psychiatric disorders. In addition to individuals with chronic mental illness, other vulnerable groups such as students may also experience amplified effects of DST due to irregular schedules and pre-existing sleep deficits, suggesting a broader population-level relevance. Providers may reduce risk by offering brief anticipatory guidance and a simple adjustment plan: Encourage patients to shift bedtime and wake time gradually (e.g., 15–20 min per day for 3–4 days before the change), protect sleep opportunity the week before and after, and reinforce core sleep hygiene (consistent schedule, limit late caffeine/alcohol, avoid long evening naps, and reduce bright/blue light in the hour before bed). Because DST may delay circadian phase by reducing morning light and extending evening light, clinicians may recommend morning bright light exposure (outdoor light soon after waking, or light therapy when appropriate) and earlier evening “wind-down” routines to support earlier melatonin onset and sleep consolidation [74]. For higher-risk patients (e.g., those with bipolar disorder, severe depression with suicidality, PTSD with insomnia/nightmares, psychosis with marked sleep disruption), clinicians may consider advising short-term symptom and sleep tracking, lower the threshold for check-ins during the 1–2 weeks post-transition, and review relapse/safety plans (including what to do if sleep drops, agitation increases, or suicidal thoughts emerge). Finally, medication routines should generally remain consistent with the new clock time, while emphasizing adherence and discussing any timing-sensitive agents (sedatives/stimulants) in advance to prevent missed doses or destabilizing self-adjustments [75].

#### 3.3.2. Policy Applications

Prior to 2023, DST was observed by roughly half of all countries; however, fewer than one-third of all countries (primarily in Europe and North America) now maintain the practice, indicating a global trend toward its discontinuation [76]. In recent years, policymakers in the United States have been debating whether to abolish seasonal DST clock changes due to health and safety concerns, with over 750 bills and resolutions considered in state legislatures [77]. Currently, 19 states have already passed laws to enact permanent DST if Congress allows, and a federal Sunshine Protection Act (which passed in the Senate in 2025 but stalled) would make DST the new, permanent standard time [77,78]. While ending seasonal clock changes has widespread support among Americans, preferences regarding which time system to adopt remain divided: Only 19% of respondents in a 2025 Gallup poll favored maintaining the current practice of clock switching, however 48% preferred permanent standard time and 24% favored permanent daylight-saving time [79].

Furthermore, sleep medicine experts and physician organizations continue to advocate for the end of DST, arguing that permanent standard time is better for public health [80]. For instance, the American Academy of Sleep Medicine warns that DST-induced misalignment may cause circadian misalignment, resulting in health and public safety-related consequences [80]. Together, policymakers’ bills, population surveys, and health organizations’ advocacy reflect broad public support for ending the twice-yearly clock change. As additional research continues to support the benefit of ending DST, legislation may increasingly prioritize circadian-aligned time policies to protect population-level mental health and public safety. Additionally, establishing whether “permanent DST” or “standard time” is the better alternative for health among vulnerable and general groups. While the review shows null effects at the population level for psychiatric hospitalizations, the consistent individual-level evidence of potential harm from circadian dysregulation through spring transitions, combined with the particular vulnerability of psychiatric populations to circadian phase advances, supports adopting perennial standard time to minimize impact for those most at risk.

### 3.4. Summary

**Figure 2:**
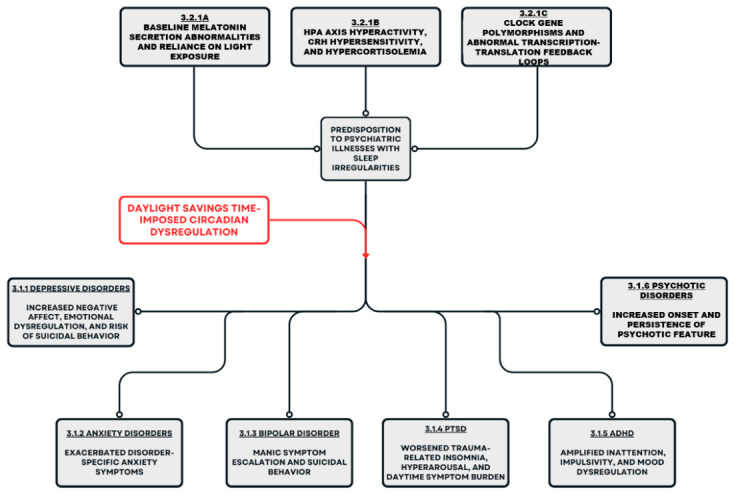
Pathways for Psychiatric Symptom Exacerbation in the Context of Daylight Savings Time.

In summary, epidemiologic, clinical, and biological evidence converge on the conclusion that DST may function as a population-wide circadian stressor capable of destabilizing sleep and biological rhythms, particularly among individuals with pre-existing psychiatric vulnerability (Figure 2). At the same time, the literature diverges in the magnitude and detectability of DST effects across diagnostic groups and outcome measures, reflecting heterogeneity in study design, populations, and levels of analysis. A key consideration in interpreting these findings is the distinction between direct and indirect evidence. While there is consistent evidence that DST transitions are associated with short-term sleep disruption, much of the evidence linking DST to psychiatric outcomes is inferred from broader literature on sleep and circadian dysregulation. As such, conclusions regarding DST-specific psychiatric risk should be interpreted cautiously. Additionally, although this narrative review primarily focuses on circadian rhythms, it is important to note that other biological rhythms, such as ultradian rhythms, may also be affected by sleep disruption and may contribute to observed cognitive and emotional changes. To address these gaps, integrated, longitudinal research that links population-level DST exposure with disorder-specific neurobiological mechanisms and clinical trials is required. Upon confirmation of effects across groups, prevention programming that aligns time studies with circadian biology can be created to mitigate mental health risk.

Importantly, direct epidemiological evidence linking DST transitions to psychiatric deterioration remains mixed, with several studies reporting null or inconsistent associations. These findings are consistent with prior studies reporting no significant association between DST transitions and psychiatric admissions or mood-related outcomes (e.g., Lahti et al., 2006 [49]; Shapiro et al., 1990 [65]). Some large-scale and registry-based analyses have not demonstrated significant increases in psychiatric hospitalizations, emergency visits, or acute mental health events following DST transitions. This variability highlights the need for cautious interpretation and underscores the distinction between direct DST-specific findings and indirect evidence derived from broader sleep and circadian research.

Another important consideration in interpreting DST-related effects is the duration and stability of post-transition adaptation. While DST is often described as producing short-term or transient disruptions, the evidence suggests a more nuanced temporal pattern. In the general population, circadian realignment and recovery of sleep patterns typically occur within several days. However, a subset of individuals may experience prolonged misalignment lasting one to two weeks, particularly following the spring transition. Among individuals with chronic mental illness or pre-existing circadian dysregulation, adaptation may be slower, less stable, or incomplete [10], potentially extending the window of vulnerability for cognitive and emotional dysregulation. This variability in adaptation underscores the importance of considering DST not as a uniform exposure, but as a temporally dynamic stressor with differential impacts across populations.

## 4. Conclusions

This comprehensive, wide-ranging review synthesizes evidence demonstrating that Daylight Saving Time (DST) represents a predictable, population-wide circadian disturbance with clinically meaningful implications for mental health, particularly among individuals with pre-existing psychiatric disorders. Findings related to the first aim of this review indicate that DST-related shifts in sleep timing and light exposure disrupt circadian alignment and sleep continuity, processes that are centrally involved in mood regulation, stress responsivity, and cognitive functioning [10,11,12]. These disrupted processes contribute to a wide range of psychiatric exacerbations in individuals with depression, anxiety, bipolar disorder, PTSD, ADHD, and psychotic disorders [22,25,35,47,58,62]. However, a more direct pathway of DST and negative psychiatric outcomes needs further exploration.

The second aim of this review focused on the neurobiological mechanisms that may underlie vulnerability to DST-related circadian disruption in psychiatric populations. Across disorders, alterations in melatonin signaling, dysregulation of the hypothalamic–pituitary–adrenal (HPA) axis, and impaired synchronization of central and genetic predispositions act as shared biological pathways through which DST could destabilize mental health [24,32,68]. These vulnerabilities are further shaped by disorder-specific mechanisms, including heightened stress reactivity in anxiety and PTSD, sleep-triggered manic episodes in bipolar disorder, and profound circadian disorganization in schizophrenia [22,30,35].

The findings of this review should also be interpreted within the context of the bidirectional relationship between sleep and mental health. While DST transitions may initially disrupt sleep and circadian alignment, the resulting changes in mood, cognition, and stress reactivity may further impair sleep quality, thereby perpetuating a feedback loop. This dynamic may be particularly relevant in individuals with pre-existing psychiatric conditions, in whom baseline vulnerabilities in sleep regulation and emotional processing may amplify the impact of even modest circadian disruptions.

An additional consideration is the potential role of substance use disorders, which were not a primary focus of the present review. Sleep disturbance and substance use share a bidirectional relationship, in which individuals may use substances such as alcohol or cannabis to manage sleep difficulties, while these substances simultaneously disrupt sleep architecture and circadian regulation. DST-related sleep disruption may therefore represent a potential, though understudied, risk factor for increased substance use or relapse. Substance use disorders represent an important and underexplored extension of DST-related circadian research, and future studies should further investigate this relationship.

From a clinical perspective, the findings included in this review support long-standing clinician recommendations to individuals with psychiatric disorders for avoiding symptom exacerbation during DST transitions [74]. From a policy perspective, the findings synthesized in this review add to a growing body of evidence suggesting that seasonal clock changes impose substantial health costs without clear benefits, supporting policymakers and physician organizations aimed at ending DST [76,78].

Collectively, these findings underscore that DST does not affect all individuals uniformly. Rather, DST interacts with underlying neurobiological and psychosocial vulnerabilities, leading to severe impacts on those with psychiatric illness. While the magnitude of DST-related effects varies across outcomes and study designs, the convergence of evidence across epidemiologic, clinical, and mechanistic domains supports the interpretation of DST as an avoidable circadian stressor with serious implications for those suffering from a mental illness.

## 5. Limitations

The overall strength of the evidence is limited by methodological constraints within the existing literature (e.g., small sample sizes, limited longitudinal data, etc.). Screening and selection of studies were conducted by a single reviewer, which may increase the risk of selection bias despite the use of predefined inclusion and exclusion criteria. Additionally, much of the research relies on cross-sectional survey studies. While these studies are well-suited to identify associations between circadian rhythm disruption and exacerbated psychiatric symptomology, they are inherently unable to establish causal relationships or temporal sequencing. This limitation is particularly salient given the bidirectional nature of many psychiatric disorders, which may be impacted by, and contribute to, circadian disruption. DST-specific psychiatric outcome studies remain sparse for several disorders, necessitating reliance on mechanistic and disorder-specific circadian research to support biological plausibility rather than direct causal attribution. Additionally, many observational studies are based on small samples, frequently drawn from single hospitals/clinics or geographic regions, thereby limiting generalizability. The widespread use of self-report instruments, such as the Pittsburgh Sleep Quality Index (PSQI), further introduces the potential for response and social desirability biases. Collectively, these factors indicate that the current evidence base is moderate in strength and constrained in its capacity to support causal inference or assess long-term and system-level effects, highlighting the need for multi-site, longitudinal, and more integrative research approaches. This review also did not specifically examine substance use disorders, which represent an important domain given their bidirectional relationship with sleep disturbance and circadian dysregulation. The exclusion of this literature limits the scope of the present synthesis and highlights an important area for future research.

In order to address some of the limitations evident in reviewed studies, future research should prioritize longitudinal, multi-site studies that explicitly examine symptom trajectories before and after DST transitions, incorporate objective circadian and sleep assessments, and identify individual-level moderators such as chronotype, medication timing, and social rhythm regularity. While many studies link DST to sleep disruption—and additional studies link sleep disruption to psychiatric symptom exacerbations—few studies directly link DST to psychiatric disturbance. Greater attention to psychiatric subpopulations and to subsyndromal outcomes may clarify why DST-related effects are detectable in some contexts, but not others. Concurrent intervention studies are needed to evaluate whether behavioral interventions, such as gradual schedule shifts, targeted light exposure, and enhanced monitoring during DST transitions, can mitigate psychiatric risk. Lastly, future policy should consider not only the effects of DST transitions, but also whether a permanent shift to standard time or DST would be more beneficial for individuals with mental illness.

By integrating a diverse body of evidence, this review demonstrates that DST-related circadian disruption is not merely a benign inconvenience, but a biologically meaningful stressor with greater consequences for vulnerable individuals with mental illness. Addressing this issue will require coordinated efforts across clinical practice, research, and policy. By integrating evidence across ecological levels, from molecular circadian mechanisms to population health outcomes, this review underscores the importance of circadian alignment as a modifiable determinant of mental health. A more effective public health approach can thus be achieved through prevention strategies that reduce psychiatric risk, including clinical vigilance, behavioral interventions, and structural policy reform.

## Figures and Tables

**Table 1:** Database-specific search strategies and the number of articles retrieved.

Database	Search Term	Number of Articles
PubMed	((“Daylight Saving Time”[tiab] OR “daylight savings time”[tiab] OR “seasonal time change”[tiab] OR “spring forward”[tiab] OR “fall back”[tiab]) OR ((“Circadian Rhythm”[Majr] OR “circadian misalignment”[tiab] OR “circadian disruption”[tiab]) AND (“Sleep Deprivation”[MeSH] OR “Sleep Wake Disorders”[MeSH] OR insomnia[tiab] OR sleep*[tiab]))) AND (“Depressive Disorder”[MeSH] OR “Bipolar Disorder”[MeSH] OR “Anxiety Disorders”[MeSH] OR “Stress Disorders, Post-Traumatic”[MeSH] OR “Attention Deficit Disorder with Hyperactivity”[MeSH] OR “Psychotic Disorders”[MeSH] OR “Schizophrenia”[MeSH]) NOT (“Shift Work”[MeSH] OR “Jet Lag Syndrome”[MeSH]) NOT (“Animals”[MeSH] NOT “Humans”[MeSH])	391
Embase	(‘daylight saving time’:ti,ab,kw OR ‘daylight savings time’:ti,ab,kw OR ‘seasonal time change’:ti,ab,kw OR ‘spring forward’:ti,ab,kw OR ‘fall back’:ti,ab,kw OR ‘circadian misalignment’:ti,ab,kw OR ‘circadian disruption’:ti,ab,kw) AND (‘depressive disorder’:ti,ab,kw OR ‘bipolar disorder’:ti,ab,kw OR ‘anxiety disorder’:ti,ab,kw OR ‘post-traumatic stress disorder’:ti,ab,kw OR ptsd:ti,ab,kw OR adhd:ti,ab,kw OR ‘psychotic disorder’:ti,ab,kw OR schizophrenia:ti,ab,kw) NOT (‘shift work’:ti,ab,kw OR ‘jet lag’:ti,ab,kw)	147
Scopus	(“daylight saving time” OR “daylight savings time” OR “seasonal time change” OR “spring forward” OR “fall back”) OR (“circadian misalignment” OR “circadian disruption”) AND (“depressive disorder” OR “bipolar disorder” OR “anxiety disorder” OR “post-traumatic stress disorder” OR PTSD OR ADHD OR “psychotic disorder” OR “schizophrenia”) AND NOT (“shift work” OR “jet lag”)	158
Web of Science	((“Daylight Saving Time” OR “daylight savings time” OR “seasonal time change”) OR (“circadian rhythm” OR “circadian misalignment” OR “circadian disruption”)) AND (“sleep deprivation” OR “sleep–wake disorder*” OR insomnia OR sleep*) AND (“depressive disorder” OR “bipolar disorder” OR “anxiety disorder*” OR “post-traumatic stress disorder” OR PTSD OR “attention deficit disorder with hyperactivity” OR ADHD OR “psychotic disorder*” OR schizophrenia) NOT (“shift work” OR “jet lag syndrome”)	860

## Data Availability

No new data were created or analyzed in this study. Data sharing is not applicable to this article.
